# The Zithromax® donation for trachoma elimination — how to apply for and manage the drug

**Published:** 2011-09

**Authors:** Lisa A Rotondo, Rachel Seligson

**Affiliations:** Deputy Director, International Trachoma Initiative, 325 Swanton Way, Decatur, GA 30030; Senior Manager, Global Logistics and Supply, Pfizer Inc.

All readers of the *Community Eye Health Journal* who are responsible for managing trachoma programmes at national, regional, or district level need to be familiar with the requirements for Zithromax® donation. The generous global donation of the antibiotic Zithromax® by Pfizer Inc. through the International Trachoma Initiative (ITI) can play an important role in the successful elimination of blinding trachoma. Therefore, the procedures required to apply for and manage the product need to be understood and followed.

Countries obtain donated Zithromax® through an application to ITI. The application is a cooperative process through which ITI, governments of trachoma-endemic countries, and other partners work together towards the national goal of eliminating blindness caused by trachoma.

Since 1998, ITI has managed Pfizer's donation of Zithromax® This drug is an effective broad-spectrum antibiotic that, when administered once annually for a consecutive number of years, works to reduce the reservoir of Chlamydia trachomatis infection in endemic communities. By distributing antibiotics in conjunction with surgical interventions, health education, and increased access to water and sanitation (known as the SAFE strategy), national trachoma programmes can stop disease transmission and progression to blindness.

Since the International Trachoma Initiative's inception, Zithromax® has been donated to 23 countries and over two hundred million doses have been administered. Careful planning is required to ensure that national programmes receive the right quantity of drugs in a timely manner and that appropriate supply chain systems are in place to manage the product when it arrives in the country where it will be used.

## Application

The Zithromax® application process starts nearly eighteen months before the drug arrives at its destination. It requires a dialogue between the relevant ministries of health, ITI, Pfizer, and international implementing partners (non-governmental organisations). The complex supply chain begins with Pfizer's suppliers, who provide raw materials, bottles, and packaging. Pfizer then manufactures the drug and arranges transport to the endemic countries. On arrival, the government, represented by the ministry of health, is expected to clear the drugs, store them safely, manage their distribution through mass drug administration (MDA), and implement the other elements of the SAFE strategy.

**Figure:**
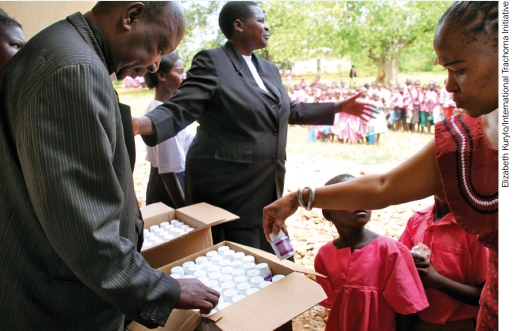
Boxes of Zithromax® ready for mass drug administration in a community. UGANDA.

ITI works directly with national trachoma programme managers who are designated by the ministry of health. ITI sends a new application form each year in November and the ministry must submit the completed application by February the following year. The application includes district-level data on past treatments, the latest trachoma prevalence data, a long-term forecast, and a current drug inventory. Despite the difficulty of forecasting in the long term, a 3-5 year needs assessment is critical to planning. The application process allows ITI to see whether there are programme gaps, allows Pfizer to plan raw material requirements, and allows the ministry of health to review their country plans and to ensure that partners are available to assist with all aspects of the SAFE strategy.

Between February and April, ITI analyses the data on drug requirements from all existing countries and new country applicants. Prior to the World Health Organization (WHO) annual trachoma alliance meeting (GET2020) in April, each country representative meets with members of ITI, Pfizer, WHO, and other implementing partners and key stakeholders in order to review the plan and produce a forecast of Zithromax requirements. This meeting also provides an opportunity to assess partner support for SAFE strategy implementation and to review baseline and impact prevalence surveys.

From these meetings, Pfizer can determine the overall drug requirements. The company can then plan manufacturing capacity for the following year, taking into account other business requirements. Any missing or additional data required for the applications is followed up by ITI in May.

In June, a final Zithromax® application is reviewed by the Trachoma Expert Committee (TEC), an independent body of international experts in public health, ophthalmology, blindness prevention, and the SAFE Strategy. TEC members provide guidance to ITI on strategic, technical and operational issues during two semiannual meetings. Based on the TEC recommendations, each ministry of health applying for Zithromax® is sent either a memorandum of understanding (MOU) for the following year detailing the quantity of Zithromax® and the districts approved for distribution, or a letter explaining why the country application was unsuccessful with suggestions to help the country to meet requirements in the future.

Once the MOU has been signed by the ministry and returned to ITI, delivery of Zithromax® is scheduled for approximately one to two months prior to the time when MDA is to take place. For countries that are already involved and that have received Zithromax® in past years, there needs to be an updated inventory of any drugs already in the country, including those with a short shelf life and those about to expire. On the basis of this information, adjustments to the donation forecast are made.

## Supply chain

Currently, Zithromax® is manufactured in two formulations, pediatric powder for oral suspension for children ages six months to five years, and tablets for people over five years of age. The product is manufactured at various sites in Europe or the USA. Once manufactured, the product is stored in one of Pfizer's European warehouse locations where the goods await allocation to recipient countries. Due to the large size of each shipment (hundreds of pallets for each country), several airplane loads may be required. Upon arrival in the country, the ministry of health must ensure customs clearance and transport to central medical stores and to regional warehouses in preparation for MDA. All charges are the responsibility of the ministry of health. A detailed description of best practices for in-country supply chain can be found in “Zithromax® in the Elimination of Blinding Trachoma: A Program Manager's Guide” at **www.trachoma.org/guides-and-manuals**

## Countries seeking Zithromax®

The Zithromax® donation is available only to governments of poor endemic countries for the elimination of blinding trachoma. Countries that have been approved for the donation may use it for MDA, administration after trichiasis surgery, or as part of surveillance or prevalence surveys, in addition to special pre-approved uses by ITI and the TEC.

National ownership and proven commitment to the elimination goal is key for a successful application and continued donation of Zithromax® The requirements for Zithromax® donation (including evidence of need, training of health care staff, SAFE strategy implementation, effective distribution strategies, and monitoring and evaluation) may be demanding for under-resourced and overstretched national health programmes. However, they are necessary to manage and monitor the Zithromax® donation to aid in the elimination of this painful and blinding disease.

